# Genome-wide characterization of the sugar transporter protein family identifies candidate genes for bacterial wilt resistance breeding in tobacco

**DOI:** 10.3389/fpls.2026.1937657

**Published:** 2026-08-31

**Authors:** Hua Xuan, Dayin Liu, Renying Xu, Lingqi Yue, Peng Wang, Pan Zhang, Jun Wu, Yushuang Guo

**Affiliations:** 1 College of Agronomy, Hunan Agricultural University, Changsha, China; 2 Yuelushan Laboratory, Changsha, China; 3 Guizhou Academy of Tobacco Science, Guiyang, China; 4 Key Laboratory of Tobacco Biological Breeding in Mountainous Ecological Zones of China National Tobacco Corporation, Guiyang, China; 5 Guizhou Provincial Key Laboratory for Tobacco Quality Improvement and Efficiency Enhancement, Guiyang, China

**Keywords:** disease resistance, NtSTP gene family, R. solanacearum, systematic evolution, tandem duplication

## Abstract

Sugar transporter proteins (STPs) play pivotal roles in hexose allocation and plant stress responses. However, systematic characterization of the STP family in tobacco (*Nicotiana tabacum*) and its involvement in *Ralstonia solanacearum* resistance remains unclear. In this study, 37 *NtSTP* genes were identified and classified into six groups, with Group VI being the most conserved and Group V exhibiting dicot-specific expansion. Gene structure and conserved motif analyses revealed that most NtSTP members possess the typical MFS_STP domain, although variations in exon–intron organization and motif composition suggested functional divergence. Tandem duplication (TD) served as the primary driver of *NtSTP* family expansion, and Ka/Ks values of all paralogous pairs were less than 1, indicative of purifying selection. Promoter cis-element analysis revealed a complex regulatory network involving hormone signaling (ABA, JA, SA, GA, ET), stress responses, and light signaling. RT-qPCR expression profiling revealed that ten *NtSTP* genes (*NtSTP1*, *5*, *7*, *21*, *22*, *24*, 26, *27*, *28*, and *29*) exhibited significant transcriptional upregulation upon *R. solanacearum* infection. Specifically, *NtSTP5*, *NtSTP7*, *NtSTP21*, *NtSTP22*, *NtSTP24*, *NtSTP26*, and *NtSTP27* peaked at 12 h post-inoculation (hpi), whereas *NtSTP1*, *NtSTP28*, and *NtSTP29* reached their highest expression levels at 24 hpi. By contrast, *NtSTP6*, *NtSTP13*, and *NtSTP30* displayed reduced expression upon *R. solanacearum* infection. These expression patterns indicate functional diversification within the *NtSTP* family and imply that these members may be transcriptionally modulated during plant responses to *R. solanacearum*. The present work provides preliminary and valuable candidate gene resources that may facilitate future disease resistance breeding programs in tobacco.

## Introduction

Sugar transporter proteins (STPs) constitute a major class of hexose transporters that facilitate cellular hexose uptake by utilizing the proton motive force generated by plasma membrane H^+^-ATPases (Doidy et al., 2012; Liu et al., 2025a). In plants, since most hexoses are derived from sucrose catabolism and the number of sucrose transporter families is considerably smaller than that of monosaccharide transporter families, STPs play dominant roles in sugar transport and allocation (Büttner, 2010; Liu et al., 2025a). Accumulating evidence indicates that *STP* genes are widely involved in sugar partitioning, plant growth and development, as well as responses to various biotic and abiotic stresses (Bavnhøj et al., 2021; Huai et al., 2022; Chen et al., 2024a).

To date, comprehensive genome-wide identification of *STP* families has been performed across various plant species, revealing 14 members in *Arabidopsis thaliana* (Büttner, 2010), 28 in *Oryza sativa* (Toyofuku et al., 2000), 81 in *Triticum aestivum* (Liu et al., 2021a), and multiple homologs in *Vitis vinifera* (Afoufa-Bastien et al., 2010) and *Solanum lycopersicum* (Reuscher et al., 2014). While most STPs exhibit broad substrate specificity, transporting glucose, galactose, fructose, and mannose (Büttner, 2007, 2010; Julius et al., 2017). In addition to facilitating the transport of sugars across the cell membrane into the storage cells, the STPs protein can also significantly influence the development and stress resistance of plants by regulating the distribution and accumulation of soluble sugars within the plant (Julius et al., 2017; Liu et al., 2018).

In *A. thaliana*, distinct *AtSTP* genes are induced by pathogen infection, abiotic stress, and phytohormone signals. For instance, *AtSTP1* and *AtSTP4* facilitate glucose uptake into guard cells to regulate stomatal aperture (Truernit et al., 1996; Sherson et al., 2000; Otori et al., 2019; Flütsch et al., 2020), whereas *AtSTP3*, *AtSTP4*, and *AtSTP8* are significantly upregulated upon pathogen infection (Truernit et al., 1996; Büttner, 2007; Liu et al., 2021b). Notably, *AtSTP13* enhances glucose absorption and bolsters resistance to *Botrytis cinerea*; it is also strongly induced by drought, salinity, and iron deficiency, linking sugar redistribution and programmed cell death to stress adaptation (Norholm et al., 2006; Yamada et al., 2011; Lemonnier et al., 2014).

Similar stress-associated functions have been characterized in other crops. In *O. sativa*, several *OsSTP* genes respond to salt, drought, osmotic stress, and hormonal cues (Deng et al., 2019), with *OsSTP15* specifically modulating sucrose allocation, cytokinin levels, and cellulose synthesis to affect seedling vigor (Li et al., 2024). In contrast, *TaSTP3*, *TaSTP6* and *TaSTP13* in *T. aestivum* have been implicated in susceptibility to *Puccinia striiformisf.* sp. *Tritici* (*Pst*) by facilitating sugar supply to the pathogen and suppressing host defense responses (Huai et al., 2019, 2020, 2022); however, other *TaSTP* members are induced by salt and drought, underscoring their role in abiotic stress tolerance (Liu et al., 2021a). Furthermore, *STP* genes in *Raphanus sativus* (Liu et al., 2022), *Brassica oleracea* var. *capitata* L (Zhang et al., 2019), and *Arachis hypogaea* (Xu et al., 2022) are induced by heavy metals, temperature extremes, drought, pathogens, and hormones, collectively confirming the widespread involvement of this family in plant stress adaptation and disease resistance.

*N. tabacum* is one of the most important economic crops worldwide; however, bacterial wilt caused by *R. solanacearum* severely compromises yield and quality (Niu et al., 2022). Given that sugar metabolism and transport are intimately linked to plant immune responses, understanding the role of *STPs* in disease resistance is of great agronomic significance (Huai et al., 2022; Chen et al., 2024a). Nevertheless, a systematic genome-wide analysis of the *N. tabacum STP* family remains lacking, and their specific roles in response to *R. solanacearum* infection are unknown. Herein, we identified 37 *NtSTP* genes and comprehensively analyzed their chromosomal locations, phylogenetic relationships, gene structures, conserved motifs, duplication events, and expression dynamics under bacterial wilt infection, thereby providing candidate genes for resistance breeding.

## Materials and methods

### Genome-wide identification of *N. tabacum* STP family members

The *N. tabacum* cv. Petite Havana SR1 (NtaSR1 v1.0) genome sequence, CDS, protein sequences and annotation files (GFF) were downloaded from the Phytozome v13 database (https://phytozome-next.jgi.doe.gov/) (Goodstein et al., 2012). The hidden Markov model (HMM) file of the STP conserved domain (PF00083) was obtained from the Pfam database (Mistry et al., 2021). HMMER 3.0 software was used to search the *N. tabacum* protein database with an E-value cutoff of 1×10^-5^. All candidate sequences were further verified using the Conserved Domain Database (CDD) (https://www.ncbi.nlm.nih.gov/Structure/bwrpsb/bwrpsb.cgi) and SMART database (https://smart.embl.de/) to remove sequences without complete STP conserved domains. Finally, the identified 37 *NtSTP* genes were named sequentially based on their chromosomal distribution and physical location. Briefly, chromosomes were ordered from Chr1 to Chr24. Within each chromosome, genes were sorted according to their starting positions from the chromosome short arm to long arm and numbered consecutively. This naming strategy follows standard conventions widely used in previous tobacco gene family investigations (Huang et al., 2026).

### Physicochemical property analysis

The amino acid length, molecular weight (MW), theoretical isoelectric point (pI), instability index, aliphatic index, and grand average of hydropathicity (GRAVY) of NtSTP proteins were analyzed using the Tbtools-II (Chen et al., 2023). Subcellular localization was predicted using the Plant-mPLoc online tools (http://www.csbio.sjtu.edu.cn/bioinf/plant/) (Chou and Shen, 2010).

### Phylogenetic tree construction

STP protein sequences of *N. tabacum*, *A. thaliana*, *Zea mays* and *O. sativa* were aligned using Muscle software with default parameters. A phylogenetic tree was constructed using MEGA 12.1 (Stecher et al., 2026) software based on the neighbor-joining (NJ) method with 1000 bootstrap replicates.

### Conserved motifs and gene structure analysis

Conserved motifs of NtSTP proteins were analyzed using the MEME Suite (https://meme-suite.org/meme/tools/meme) (Bailey et al., 2009) online tool with the following parameters: maximum number of motifs = 10, optimum motif width = 6–50. The exon–intron structures of *NtSTP* genes were visualized using the Tbtools-II software (Chen et al., 2023).

### Gene duplication and evolutionary pressure analysis

Tandem duplication and Whole-genome duplication (WGD) or segmental duplication events were identified using MCScan X software (Wang et al., 2012). The non-synonymous substitution rate (Ka), synonymous substitution rate (Ks) and Ka/Ks ratio were calculated using the Tbtools-II software (Chen et al., 2023) to evaluate the evolutionary selection pressure. Tandem duplicates and allopolyploidy-derived homoeologous gene pairs were distinguished according to their chromosomal locations. Homoeologous gene pairs are distributed on different chromosomes belonging to the S-subgenome and T-subgenome, respectively. Genes were defined as tandem duplicates if two paralogs were adjacent on the same chromosome, with an interval shorter than 100 kb and no unrelated annotated genes between them.

### Interspecies collinearity analysis

Collinear relationships between *N. tabacum* and four representative species (*A. thaliana*, *S. lycopersicum*, *O. sativa*, and *Z. mays*) were analyzed using MCScan X (Wang et al., 2012) and visualized using Tbtools-II software (Chen et al., 2023). The collinear pairs were used to reveal the evolutionary conservation and divergence of the STP gene family.

### Promoter cis-acting element analysis

The 2000 bp upstream sequences from the translation initiation site (ATG) of each *NtSTP* gene were extracted as promoter regions using Tbtools-II software (Chen et al., 2023). The cis-acting elements were predicted using the PlantCARE (https://bioinformatics.psb.ugent.be/webtools/plantcare/html/) (Lescot et al., 2002), online database and visualized using Tbtools-II software (Chen et al., 2023).

### Plant materials and growth conditions

*N. tabacum* cv. K326 were used in this study. Tobacco plants were grown in a greenhouse under a 16 h/8 h (light/dark) photoperiod at 25°C/22°C (light/dark), a light intensity of approximately 200 μmol m^-2^ s^-1^, and relative humidity of 60-70%. Plants were used for experiments at the 5–6 leaf stage (approximately 4–5 weeks after germination) when leaves were fully expanded.

### *R. solanacearum* growth and infection assays

*R. solanacearum* strains VGS were used in this study. Bacterial strains were stored at −80°C and cultured on BEP medium (5 g/L beef extract, 10 g/L peptone, 15 g/L NaCl, 20 g/L agar, pH 7.0) at 30°C for 2–3 days. A single colony was inoculated into liquid medium and grown at 28°C with shaking for 48 h until reaching the exponential phase. Bacterial cells were collected and resuspended in sterile water to final concentration of 1×10^8^ CFU/mL. Fully expanded leaves were inoculated using a needle-prick method with 10 μL bacterial suspension per site. Plants were maintained at 25°C under an 8 h/16 h (light/dark) photoperiod. For time-course expression analysis, inoculated leaf tissues surrounding the inoculation sites were collected at 0, 6, 12, 24, 48, and 72 h post inoculation (hpi). Samples from each treatment and time point were collected from three independent biological replicates and immediately frozen in liquid nitrogen for RNA extraction and RT-qPCR analysis.

### RNA extraction and RT-qPCR assay

The total RNA was extracted from indicated plant materials using RNA isolater total RNA extraction reagent (Vazyme, R401). Approximately 100 mg of plant tissue was used for RNA extraction. The concentration and quality of extracted RNA were determined using NanoDrop spectrophotometer and agarose gel electrophoresis. Genomic DNA contamination was removed during reverse transcription using the HiScript III RT SuperMix for qPCR (+gDNA Wiper) (Vazyme, RT101). First-strand cDNA was synthesized with 1 μg total RNA.

The quantitative real-time PCR was performed with the ChamQ universal SYBR qPCR master mix (Vazyme, Q711) in a 10 μL reaction volume. The amplification procedure consisted of an initial denaturation at 95 °C for 30 s, followed by 40 cycles of 95 °C for 10 s, 60 °C for 30 s, followed by melting curve analysis to confirm amplification specificity. The *NtUbiquitin* gene was used as an internal reference. The stability of *NtUbiquitin* expression under the experimental conditions was confirmed based on its consistent expression among samples. Three independent biological replicates were performed for each treatment, and each biological replicate was analyzed using two technical replicates. The two technical replicates were averaged and used for subsequent statistical analysis. Relative transcript levels were calculated using the 2^−ΔΔCt method. All primers are listed in **Supplementary Table 1**.

### Statistical analysis

All statistical analyses were performed using GraphPad Prism v9.0. RT-qPCR expression data are presented as mean ± standard deviation (SD). Each treatment included three independent biological replicates with two technical replicates per biological sample. Student’s unpaired two-tailed t-tests was applied to assess expression differences across different inoculation time points. Differences were considered statistically significant at p < 0.05.

## Results

### Identification and physicochemical properties of *N. tabacum* STP family proteins

**Figure 1:**
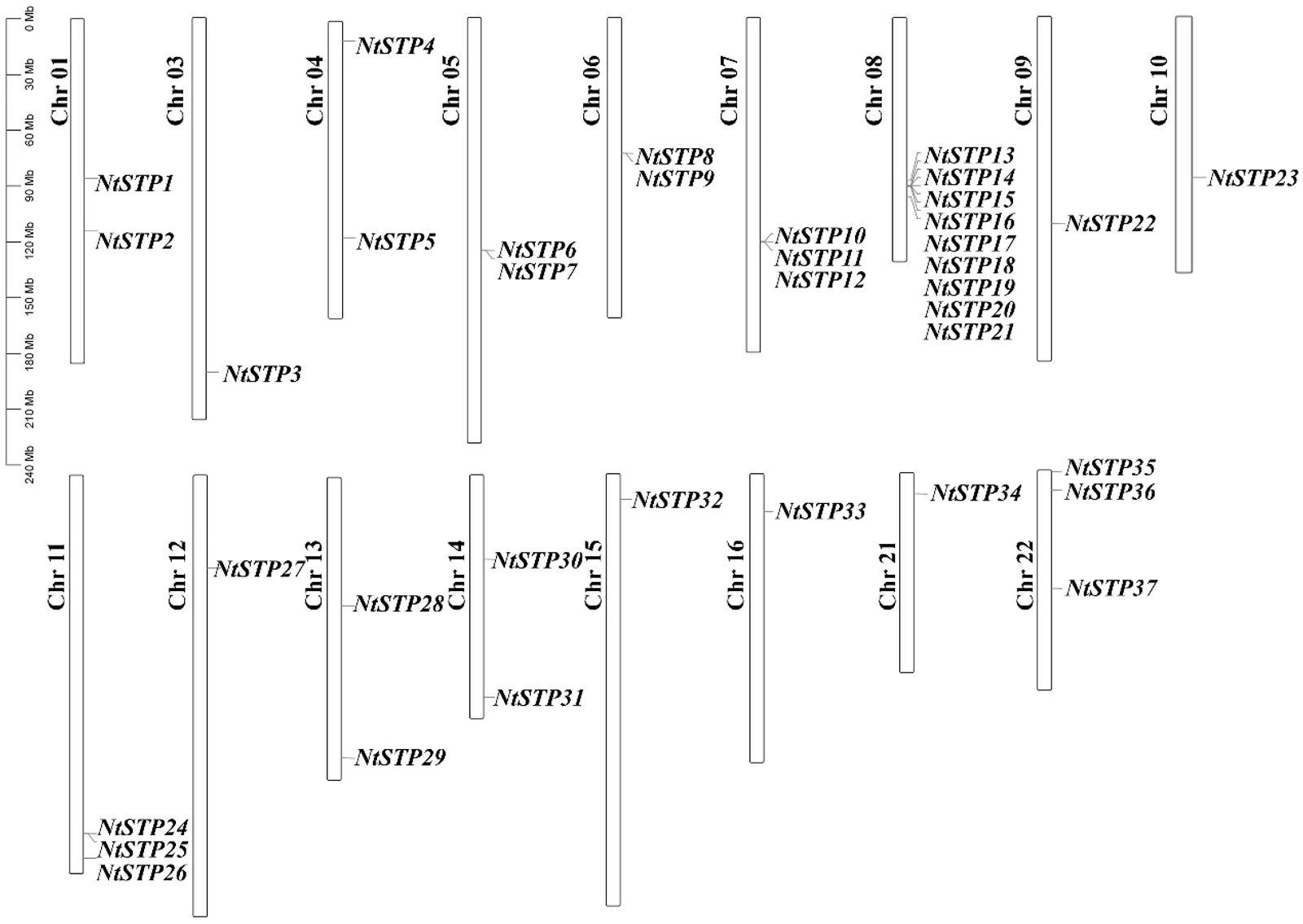
Chromosomal localization of the *N. tabacum STP* family genes.

In this study, a total of 37 *STP* genes were identified from the *N. tabacum* genome and named *NtSTP1* to *NtSTP37* according to their chromosomal distributions (**Figure 1**; **Table 1**), following common practices in plant gene family analyses (Huang et al., 2026).

**Table 1:** Physicochemical properties of *STP* family gene in *N. tabacum*.

Sequence ID	Gene name	Chr	Start	End	Number of amino acid	Molecular weight	Theoretical	Instability index	Aliphatic index	Grand average of hydropathicity
Nta01g20960.1	NtSTP1	Chr1	85821808	85823376	522	57.17	9.03	34.56	103.28	0.55
Nta01g23930.1	NtSTP2	Chr1	114151605	114159407	525	57.57	8.93	37.59	109.18	0.495
Nta03g28790.1	NtSTP3	Chr3	190702186	190706247	523	57.69	9.21	31.14	106.21	0.546
Nta04g02470.1	NtSTP4	Chr4	10635390	10639088	511	56.88	8.99	36.34	102.29	0.494
Nta04g22160.1	NtSTP5	Chr4	116420201	116424026	523	57.72	9.04	30.51	106.79	0.562
Nta05g11790.1	NtSTP6	Chr5	125289460	125293432	509	55.35	9.53	37.51	109.98	0.55
Nta05g11800.1	NtSTP7	Chr5	125299348	125302798	510	55.66	9.33	30.79	105.73	0.522
Nta06g10530.1	NtSTP8	Chr6	73017503	73020524	508	55.28	9.53	34.36	111.16	0.566
Nta06g10540.1	NtSTP9	Chr6	73021941	73025194	509	55.57	9.46	30.48	105.17	0.524
Nta07g16670.1	NtSTP10	Chr7	120373853	120375433	526	57.37	9.16	33.12	103.44	0.564
Nta07g16680.1	NtSTP11	Chr7	120496197	120497777	526	57.37	9.16	33.12	103.44	0.564
Nta07g16720.1	NtSTP12	Chr7	120859421	120861001	526	57.37	9.16	33.12	103.44	0.564
Nta08g13470.1	NtSTP13	Chr8	87333647	87335338	563	62.18	9.02	30.93	104.78	0.513
Nta08g14010.1	NtSTP14	Chr8	90245989	90247557	522	57.04	9.1	36.07	103.47	0.568
Nta08g14030.1	NtSTP15	Chr8	90387923	90389491	522	57.04	9.1	36.07	103.47	0.568
Nta08g14040.1	NtSTP16	Chr8	90397953	90399521	522	57.04	9.1	36.07	103.47	0.568
Nta08g14050.1	NtSTP17	Chr8	90409319	90410887	522	57.04	9.1	36.07	103.47	0.568
Nta08g14060.1	NtSTP18	Chr8	90420683	90422251	522	57.04	9.1	36.07	103.47	0.568
Nta08g14090.1	NtSTP19	Chr8	90609282	90610667	461	50.77	9.37	37.96	107.42	0.584
Nta08g14100.1	NtSTP20	Chr8	90829858	90831243	461	50.77	9.37	37.96	107.42	0.584
Nta08g15230.1	NtSTP21	Chr8	96267142	96270508	508	54.70	9.31	39.45	104.29	0.605
Nta09g16090.1	NtSTP22	Chr9	111535335	111539665	512	56.05	8.89	35.02	104.41	0.485
Nta10g13530.1	NtSTP23	Chr10	85971383	85976264	516	56.37	8.8	33.97	104.75	0.494
Nta11g26910.1	NtSTP24	Chr11	192470293	192471569	377	40.92	8.66	36.3	107.85	0.568
Nta11g26920.1	NtSTP25	Chr11	192510518	192511561	347	37.35	9.21	31.03	103.98	0.488
Nta11g29460.1	NtSTP26	Chr11	205928555	205933268	513	56.18	8.88	36.44	111.81	0.551
Nta12g11570.1	NtSTP27	Chr12	49949399	49952191	490	53.98	8.93	34.53	111.02	0.639
Nta13g11640.1	NtSTP28	Chr13	69131642	69134699	508	54.95	8.93	26.93	107.11	0.621
Nta13g20840.1	NtSTP29	Chr13	151023066	151028570	514	56.73	9.61	40.96	107.59	0.444
Nta14g10560.1	NtSTP30	Chr14	45396194	45398779	508	54.89	8.93	26.93	107.3	0.628
Nta14g19190.1	NtSTP31	Chr14	119538990	119544495	514	56.74	9.61	41.15	108.15	0.447
Nta15g03710.1	NtSTP32	Chr15	13755830	13758741	446	49.55	8.89	46.29	104.01	0.45
Nta16g03870.1	NtSTP33	Chr16	20404998	20407687	472	52.19	8.48	45.17	104.09	0.474
Nta21g02760.1	NtSTP34	Chr21	11406404	11410548	526	58.30	9.08	32.08	103.76	0.506
Nta22g00330.1	NtSTP35	Chr22	934884	938485	487	53.60	9.17	32.6	108.15	0.627
Nta22g02630.1	NtSTP36	Chr22	10825909	10830703	529	58.43	9.05	30.71	105.05	0.512
Nta22g08860.1	NtSTP37	Chr22	63558712	63564915	523	57.41	9.12	37.28	109.6	0.503

**Figure 2:**
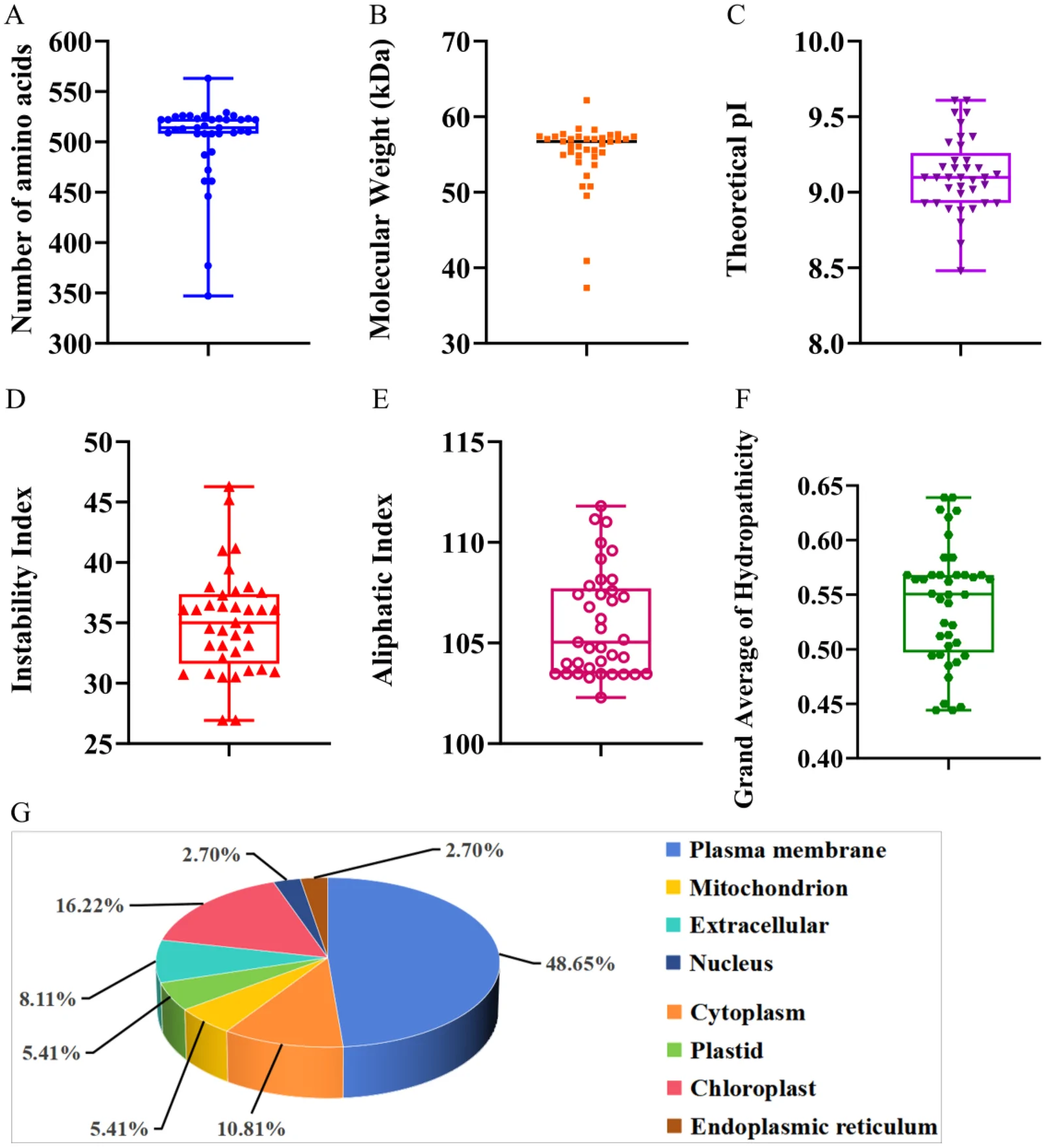
Physicochemical Properties of NtSTP Proteins. **(A)** Amino acid sequence length distribution of *N. tabacum* STP proteins. **(B)** Molecular weight (MW) distribution. **(C)** Theoretical isoelectric point (pI) values. **(D)** Instability index. **(E)** Aliphatic index. **(F)** Grand average of hydropathicity (GRAVY) values. **(G)** Subcellular localization prediction of NtSTP proteins.

The physicochemical properties of the 37 identified NtSTP proteins were systematically characterized using Tbtools-II software (Chen et al., 2023). The results showed that the length of the amino acids varied from 347 residues in NtSTP25 to 563 residues in NtSTP13 (**Figure 2A**), corresponding to molecular weights between 37.35 kDa (NtSTP25) and 62.18 kDa (NtSTP13) (**Figure 2B**). Furthermore, the theoretical isoelectric points (pI) exhibited a range from 8.48 (NtSTP33) to 9.61 (NtSTP29), indicating that all NtSTP proteins are alkaline (pI > 7) (**Figure 2C**). Analysis of the instability index revealed that 33 proteins (89.19%) displayed values below 40, suggesting a propensity for stable structural conformations (**Figure 2D**). Consistent with this, the aliphatic indices ranged from 102.29 to 111.81 (all > 100), implying thermostability and stable structural conformation across the family (**Figure 2E**). However, evaluation of the grand average of hydropathicity (GRAVY) showed that all proteins were hydrophobic properties (**Figure 2F**). These pronounced differences in intrinsic physicochemical parameters among *N. tabacum* STP proteins imply significant functional diversification within this gene family.

Determining the subcellular localization of transporters is critical for elucidating their biological functions. In silico prediction using Plant-mPLoc suggested that 48.65% (18/37) of NtSTP proteins are potentially likely to be localized to the plasma membrane (**Figure 2G**). Although this computational result aligns with the canonical function of STPs as plasma membrane hexose transporters, we emphasize that these localization patterns remain predictive and lack experimental verification. As highlighted in previous studies, prediction algorithms based on amino acid sequences have inherent uncertainty and may deviate from the real subcellular distribution of proteins (Mia et al., 2026a, 2026). Therefore, *in vivo* fluorescence imaging assays such as GFP fusion are needed in follow-up research to validate the actual localization of NtSTP transporters.

### Phylogenetic analysis of NtSTP family

**Figure 3:**
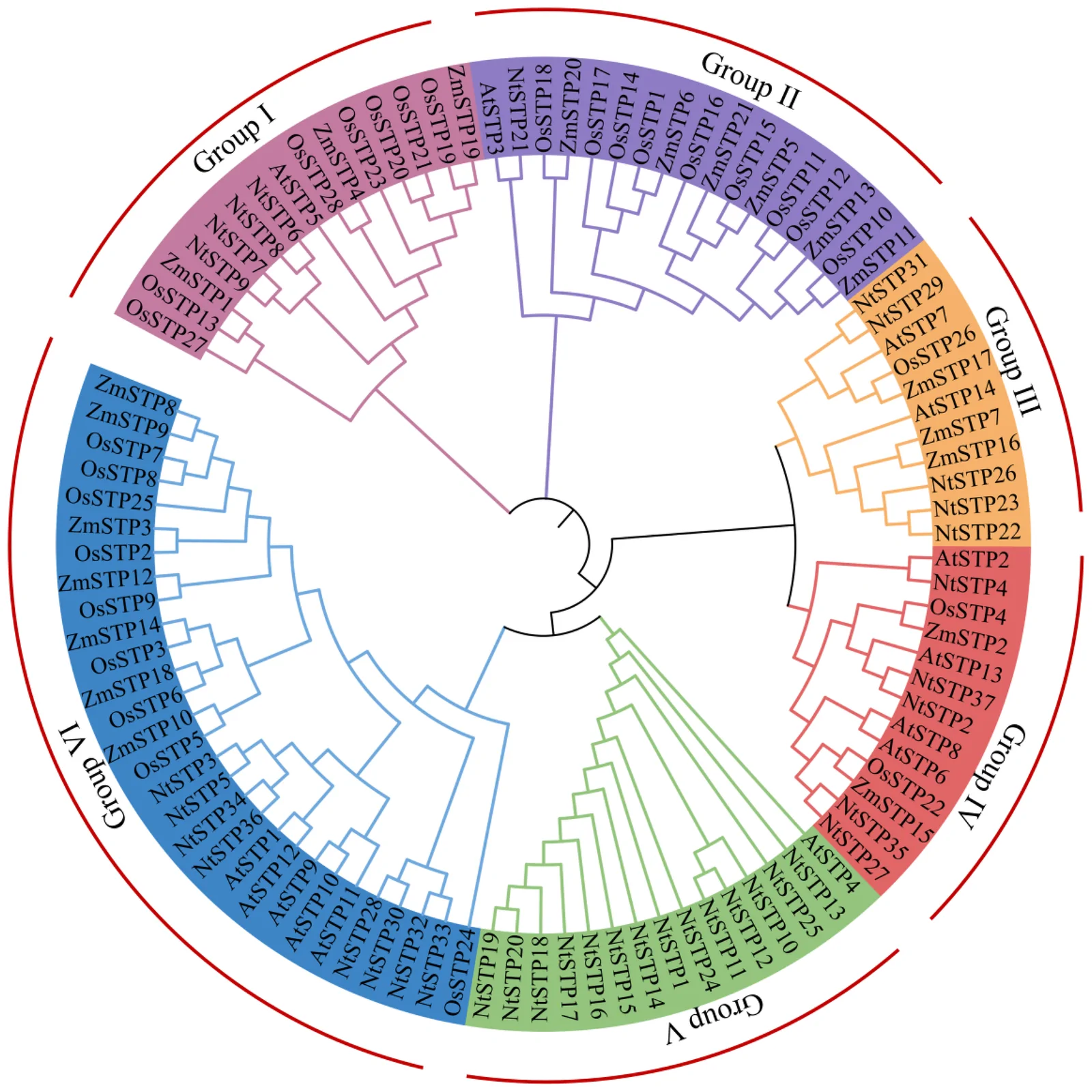
The phylogenetic trees were generated of 37 NtSTP, 28 OsSTP, 21 ZmSTP and 14 AtSTP proteins by MEGA 12.1 (method: Neighbor-Joining; parameter: bootstrap values of 1000 replicates). Magenta, purple, orange, red, green and blue represents Group I, Group II, Group III, Group IV, Group V and Group VI, respectively.

To explore the evolutionary relationships of the STP family, full-length protein sequences from *N. tabacum*, *A. thaliana*, *O. sativa* and *Z. mays* were employed to construct phylogenetic trees. Two tree-building algorithms (NJ and ML) were applied separately. The resulting topologies exhibited strong congruence, without notable conflicts in the grouping of STP homologs, subfamily classification and species clustering (**Figure 3**; **Supplementary Figure 1**). As shown in **Figure 3**, all STP proteins were clustered into six independent clades (Groups I-VI). Notably, NtSTP proteins were distributed across all six clades, with the majority clustering closely with AtSTPs, indicating a closer evolutionary affinity between *N. tabacum* STPs and *A. thaliana* than with monocot species (*O. sativa* and *Z. mays*). Statistical analysis of member distribution revealed significant disparities in clade composition (**Figure 3**). Group VI constituted the largest clade, including 5 AtSTPs, 9 OsSTPs, 7 ZmSTPs, and 8 NtSTPs, which accounted for approximately 29% of the total 100 STP proteins, suggesting that Group VI is the most conserved subfamily and may be involved in core biological processes shared by monocots and dicots. Conversely, Group V exhibited a striking species-specific expansion, containing 14 NtSTPs and 1 AtSTP, but no OsSTPs and ZmSTPs, implying lineage-specific functional divergence in dicots. Group II was the second largest, dominated by monocot STPs (9 OsSTPs and 6 ZmSTPs), while Groups I, III, and IV contained mixed distributions of members from all four species (**Figure 3**).

The differential distribution patterns imply varying evolutionary pressures among clades. The dominance of monocot STPs in Group II suggests functional expansion potentially linked to the distinct growth architectures and environmental adaptations of monocotyledonous plants. In contrast, the balanced representation in Groups IV and VI implies functional conservation across angiosperms. Intriguingly, the dicot-specific nature of Group V suggests that this clade may have undergone neofunctionalization or subfunctionalization during the evolution of *N. tabacum* and *A. thaliana*, potentially endowing it with unique functional characteristics. Collectively, these results provide valuable insights into the evolutionary origins, functional differentiation, and species-specific adaptations of the NtSTP.

### Conserved motifs and gene structure analysis

**Figure 4:**
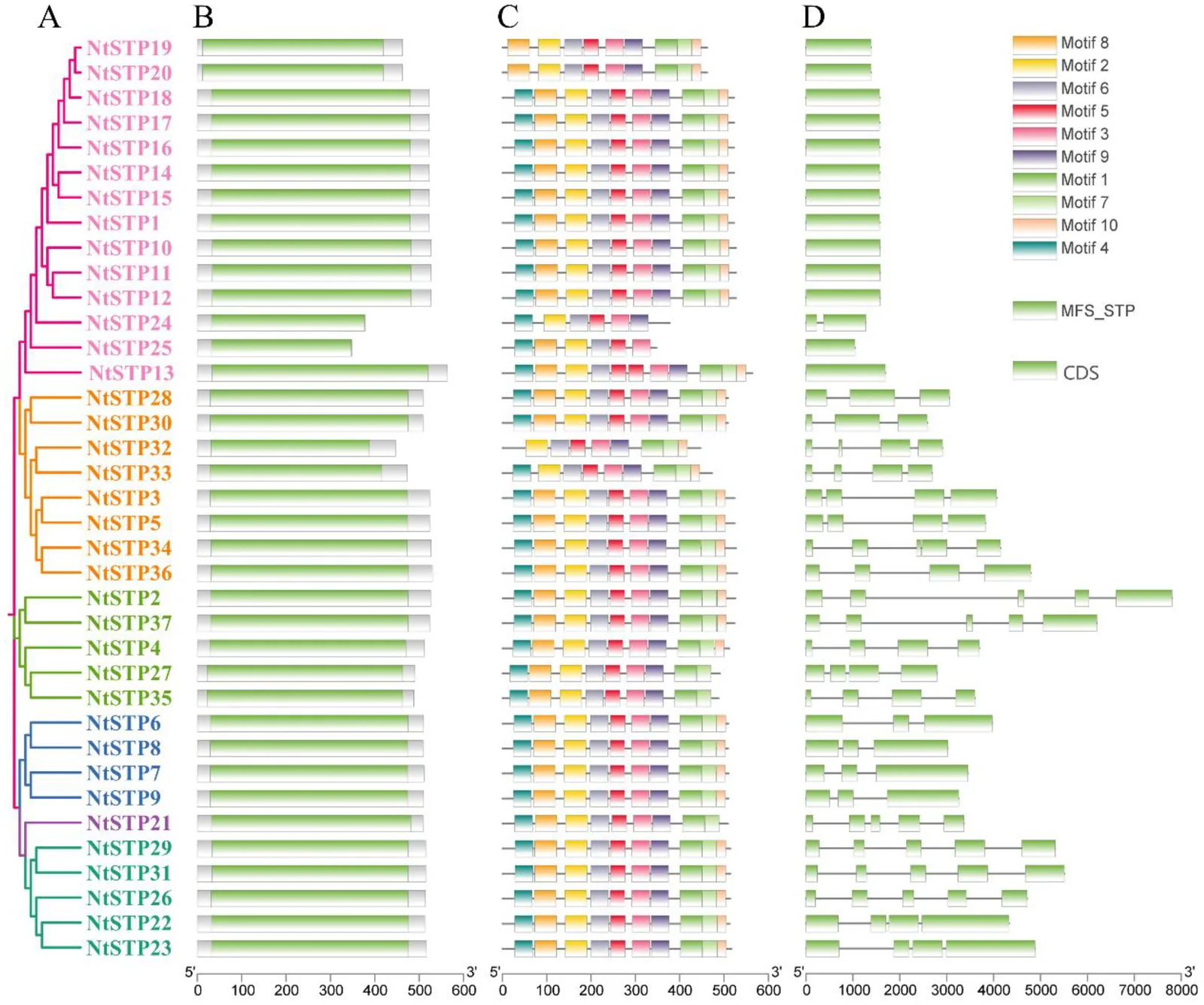
Phylogenetic tree, conserved motif, conserved domain and gene structure were analyzed of NtSTP. **(A)** Phylogenetic tree of NtSTP proteins. **(B)** Schematic diagram of the MFS_STP domain architecture. **(C)** Distribution of ten conserved motifs identified by MEME. Different colored boxes represent different motifs. **(D)** Exon-intron structure of *NtSTP* genes.

To characterize the structural features of NtSTP proteins, conserved domain prediction was implemented using NCBI-CDD. Our results showed that all 37 NtSTP proteins contained the conserved typical MFS_STP domain (**Figures 4A, B**), confirming the structural authenticity of the identified *NtSTP* family genes.

To elucidate the evolutionary diversity and functional differentiation of the *NtSTP* family, conserved motifs were identified using the MEME. Ten distinct motifs (Motifs 1-10) were detected across the 37 NtSTP proteins (**Figure 4C**). Most members contained the full complement of these motifs, indicating that Motifs 1–10 represent the core structural signature essential for the fundamental transport function of STPs. Furthermore, members within the same phylogenetic clade shared highly similar motif compositions, reinforcing the phylogenetic classification and suggesting functional conservation within subgroups. Notably, variations in motif composition were observed, likely reflecting functional divergence. While Group I and Group III members harbored all ten motifs, Group IV members generally lacked Motif 10 (except NtSTP27 and NtSTP35) (**Figure 4C**). In Group VI, specific deletions of Motifs 2 and/or 4 were evident in NtSTP32 and NtSTP33. Group V exhibited the most pronounced divergence; for instance, NtSTP19, NtSTP24, and NtSTP25 displayed substantial motif loss, retaining only partial core motifs. Such differential motif loss may enable these proteins to adapt to specific biological processes.

To further explore the structural diversity of *NtSTP* genes, exon-intron distribution patterns were analyzed using TBtools software (**Figure 4D**). Consistent with the motif analysis, significant structural diversification was observed. Group V genes possessed a remarkably simplified structure, characterized by a single exon (intronless), with NtSTP24 being the sole exception containing two exons (**Figure 4D**). In contrast, members of the other subgroups exhibited more complex architectures, with 3 to 5 exons. Intriguingly, this structural diversification, intronless in Group V versus intron-rich in other groups, likely represents a key evolutionary driver allowing the NtSTP family to adapt to diverse biological processes, including growth, development, and stress responses in *N. tabacum*.

### Gene duplication and Ka/Ks analysis

**Figure 5:**
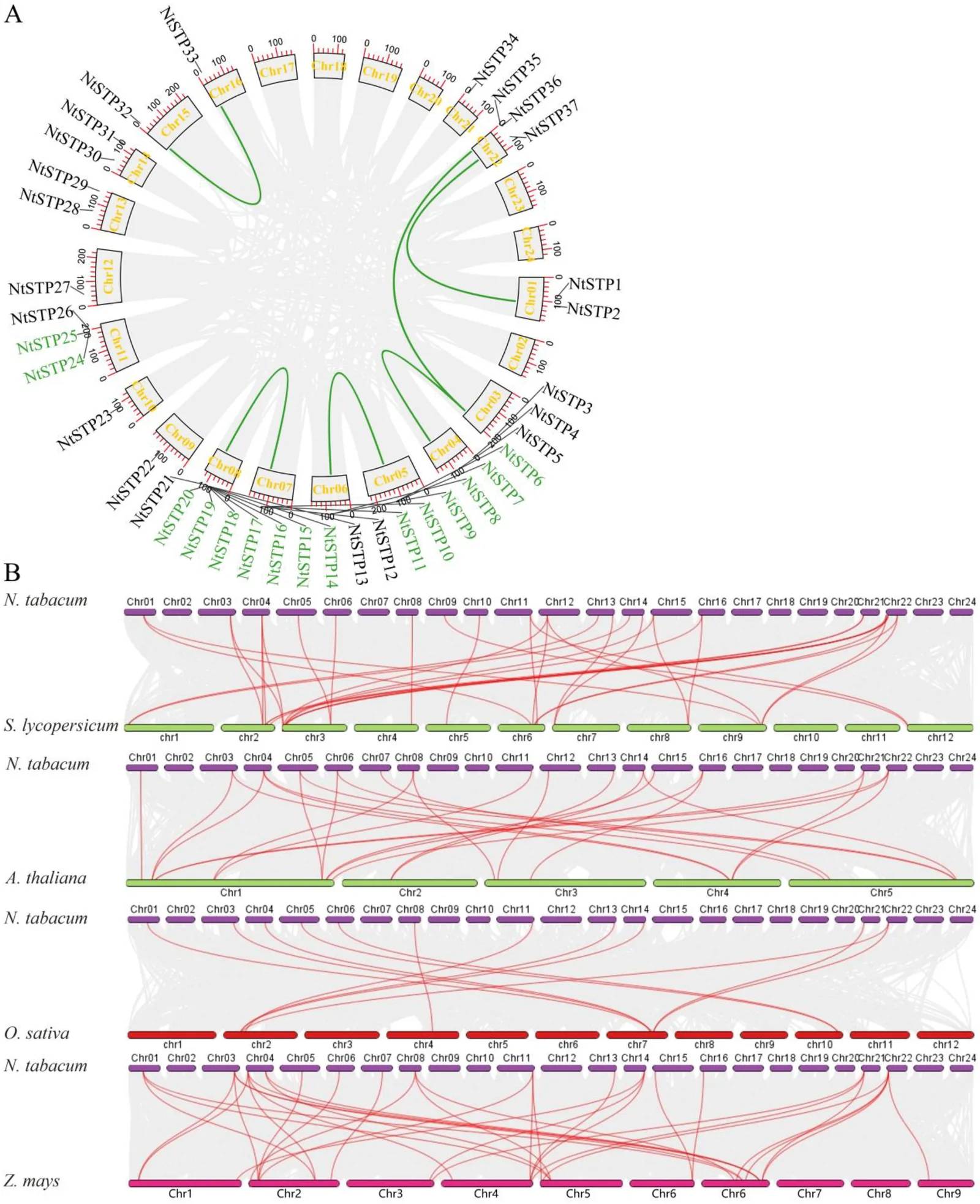
Gene duplication and synteny analysis of the *NtSTP* gene family. **(A)** Gene duplication events of *NtSTP* genes. Whole-genome duplication (WGD) events are indicated by green lines, and tandem duplicated genes are labeled with green gene IDs. **(B)** Interspecific synteny analysis between *N. tabacum* and *A. thaliana*, *O. sativa* and *S. lycopersicum*.

Gene duplication is a major driving force shaping the expansion and functional diversification of multi-gene families (Xue et al., 2024). To elucidate the evolutionary dynamics of the *NtSTP* family, intraspecific collinearity analysis was performed using MCScan X. The results revealed that tandem duplication (TD) played a predominant role in the expansion of *NtSTP* genes (**Figure 5A**). These genes exhibited a distinct clustered distribution across the chromosomes, a hallmark of TD events. This localized amplification generates highly homologous gene copies that may undergo subfunctionalization or neofunctionalization, thereby enhancing adaptability to environmental stresses such as pathogen invasion or drought.

To further understand the selective pressures acting on duplicated genes, the non-synonymous (Ka) to synonymous (Ks) substitution rate ratios were calculated. The Ka/Ks ratios of all collinear *NtSTP* gene pairs were consistently less than 1 (**Table 2**), indicating that the evolution of the *NtSTP* family has been governed by strong purifying selection. Furthermore, the TD and WGD occurred between 0.17 to 76.48 million years ago (Mya) (**Table 2**).

**Table 2:** Ka/Ks and Divergence time estimation analysis of *NtSTP* genes.

Seq_1	Seq_2	Ka	Ks	Ka_Ks	mya	Duplicate event
NtSTP6	NtSTP7	0.1890	1.3919	0.1358	76.4775	Tandem duplication
NtSTP8	NtSTP9	0.1942	1.3775	0.1410	75.6886	Tandem duplication
NtSTP10	NtSTP11	0.0000	0.0000	0.0000	0.0000	Tandem duplication
NtSTP14	NtSTP15	0.0027	0.0187	0.1428	1.0287	Tandem duplication
NtSTP15	NtSTP16	0.0000	0.0000	0.0000	0.0000	Tandem duplication
NtSTP16	NtSTP17	0.0000	0.0000	0.0000	0.0000	Tandem duplication
NtSTP17	NtSTP18	0.0000	0.0000	0.0000	0.0000	Tandem duplication
NtSTP19	NtSTP20	0.0000	0.0031	0.0000	0.1703	Tandem duplication
NtSTP24	NtSTP25	0.0345	0.0684	0.5041	3.7565	Tandem duplication
NtSTP2	NtSTP37	0.0076	0.0956	0.0795	5.2547	Whole genome duplication
NtSTP3	NtSTP5	0.0067	0.1009	0.0665	5.5437	Whole genome duplication
NtSTP3	NtSTP36	0.0727	0.5666	0.1284	31.1314	Whole genome duplication
NtSTP6	NtSTP8	0.0087	0.0986	0.0882	5.4201	Whole genome duplication
NtSTP7	NtSTP9	0.0156	0.1198	0.1302	6.5827	Whole genome duplication
NtSTP10	NtSTP15	0.0101	0.0863	0.1168	4.7430	Whole genome duplication
NtSTP32	NtSTP33	0.0213	0.1530	0.1390	8.4088	Whole genome duplication

Furthermore, interspecific collinearity analysis was conducted to compare the *N. tabacum STP* genes with orthologs from *A. thaliana*, *O. sativa*, and *S. lycopersicum*. The results showed extensive collinear relationships between *NtSTP* and *S. lycopersicum STP* genes, with numerous orthologous gene pairs identified (**Figure 5B**). In contrast, collinearity between *N. tabacum* and *O. sativa STP* genes was relatively limited. These findings indicate that *N. tabacum* shares a closer evolutionary relationship with dicotyledonous *S. lycopersicum*, and their *STP* homologs may retain conserved biological functions inherited from common ancestral genes. Collectively, interspecific collinearity analysis provides valuable evolutionary clues for the functional prediction and annotation of *NtSTP* genes based on the well-characterized functions of homologous genes in model plants.

### Promoter cis-acting element analysis

**Figure 6:**
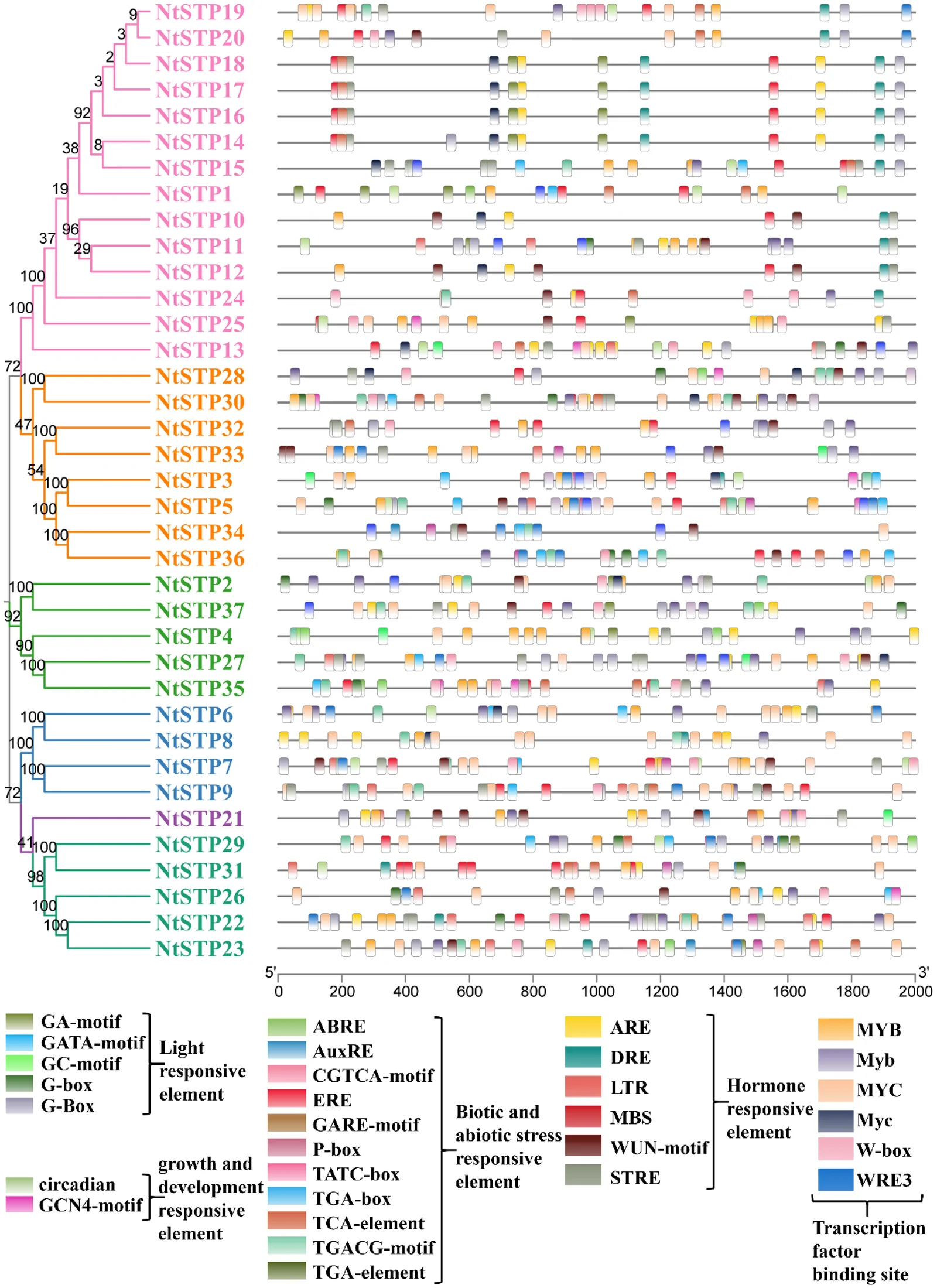
The cis-elements were predicted in 2000 bp promoter sequences of *NtSTP* genes. The Cis-elements was analyzed of *NtSTP* genes by Plantcare.

To elucidate the transcriptional regulatory networks governing the *NtSTP* gene family, the 2000-bp upstream regions of the translation start sites were analyzed as promoters using the PlantCARE database (**Figure 6**). The results revealed a complex repertoire of cis-regulatory elements, which were categorized into five major groups: hormone-responsive elements, stress-responsive elements, light-responsive elements, growth and development regulators, and transcription factor binding sites.

Specifically, hormone-responsive elements were the most prevalent, comprising six functional subtypes mediating signals from abscisic acid (ABRE), methyl jasmonate (CGTCA/TGACG motifs), auxin (AuxRE, TGA-box), gibberellin (GARE, P-box), salicylic acid (TCA-element), and ethylene (ERE). Furthermore, the promoters were enriched with elements related to abiotic stress adaptation, including anaerobic induction (ARE), low-temperature responsiveness (LTR), drought stress (MBS), and general defense (TC-rich repeats, WUN-motif). Notably, light-responsive elements (e.g., G-box, GT1-motif) and development-related motifs (e.g., GCN4, circadian) were also identified, along with numerous binding sites for key transcription factors such as MYB, MYC, and W-box.

In summary, the promoter architecture of the *NtSTP* family is characterized by a sophisticated array of cis-elements involved in hormone signaling, stress response, and photomorphogenesis. This intricate regulatory landscape suggests that *NtSTP* genes are subject to multi-layered transcriptional control, enabling their diverse roles in plant adaptation and growth.

### Expression patterns of *NtSTP* genes under *R. solanacearum* infection

**Figure 7:**
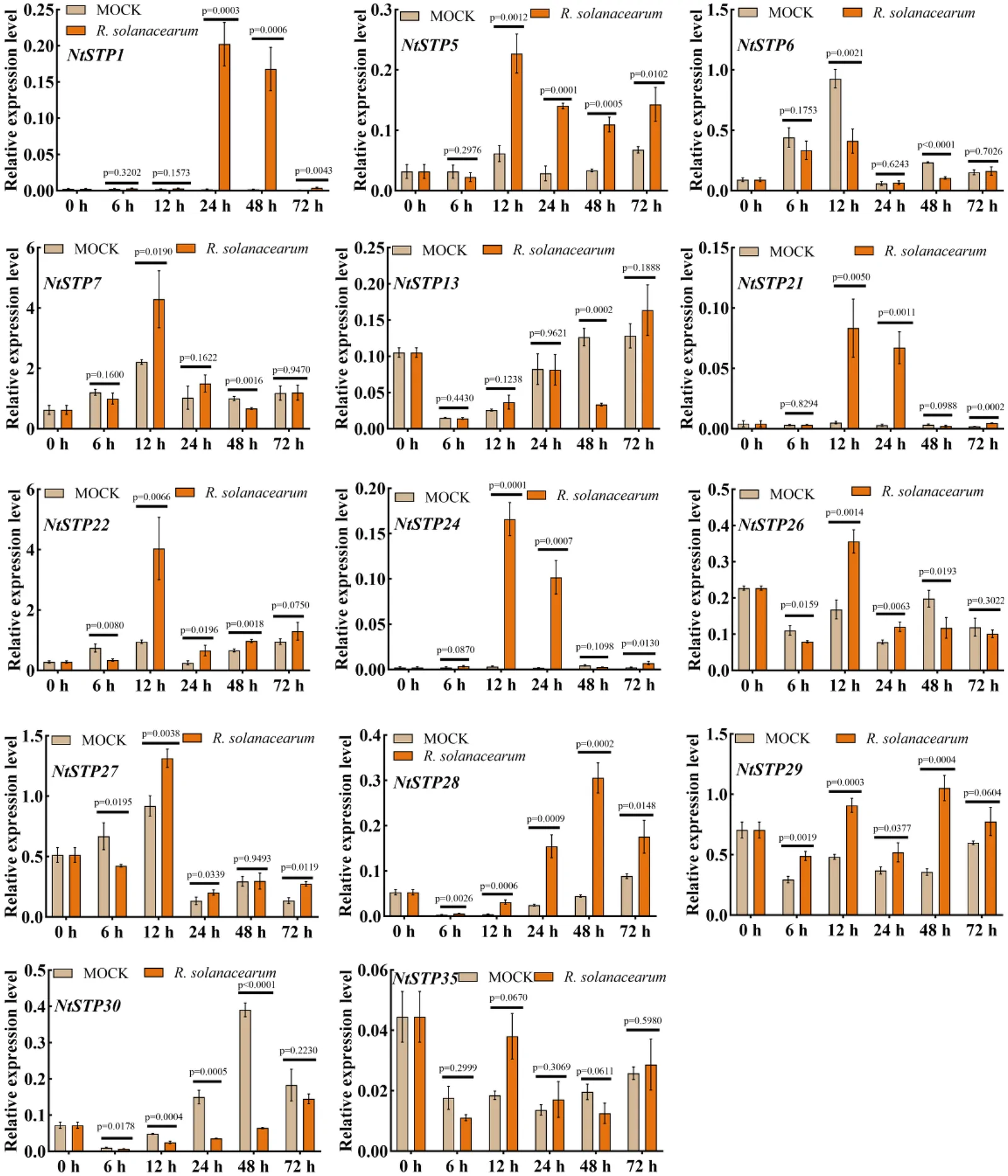
Time-course expression analysis of *NtSTP* genes in response to *R. solanacearum* infection. Relative transcript levels were quantified by RT-qPCR at 0, 6, 12, 24, 48, and 72 h post-inoculation (hpi) following *R. solanacearum* infection or mock treatment with sterile water. Data are presented as mean ± SD from three independent biological replicates, with each biological replicate analyzed using two technical replicates. The two technical replicates were averaged before statistical analysis. Statistical significance was determined by student’s unpaired two-tailed t-tests. Differences were considered statistically significant at p < 0.05.

*N. tabacum* is an economically important cash crop worldwide, while bacterial wilt disease caused by the soil-borne pathogen *R. solanacearum* severely impairs *N. tabacum* yield and quality in major planting areas. Accumulating evidence has revealed that *STP* family genes participate in plant immune responses against pathogen invasion. To explore the potential responses of *NtSTP* genes under *R. solanacearum* stress, 14 *NtSTP* members were selected for RT-qPCR analysis to characterize expression profiles at 0, 6, 12, 24, 48, and 72 h post *R. solanacearum* inoculation. Using a stratified sampling strategy, genes were picked from each of the six phylogenetic subgroups to survey expression variation across the *NtSTP* family. Our results revealed varied expression patterns of *NtSTP* genes under *R. solanacearum* infection (**Figure 7**). Ten genes (*NtSTP1*, *NtSTP5*, *NtSTP7*, *NtSTP21*, *NtSTP22*, *NtSTP24*, *NtSTP26*, *NtSTP27*, *NtSTP28*, and *NtSTP29*) were significantly induced by *R. solanacearum*. *NtSTP5*, *NtSTP7*, *NtSTP21*, *NtSTP22*, *NtSTP24*, *NtSTP26*, and *NtSTP27* peaked at 12 h post inoculation, whereas *NtSTP1*, *NtSTP28*, and *NtSTP29* achieved maximum expression at 24 hpi. Notably, *NtSTP28* was significantly up-regulated at all examined time points (6, 12, 24, 48, and 72 hpi). In contrast, *NtSTP22*, *NtSTP26*, and *NtSTP27* exhibited a transient down-regulation at 6 hpi, followed by induction at later stages. Conversely, *NtSTP6* and *NtSTP30* showed consistent down-regulation throughout the infection period. Overall, *NtSTP* family members exhibit different temporal expression characteristics under bacterial wilt stress, suggestive of functional divergence during pathogen exposure. These expression changes identify *NtSTP1/5/7/21/22/24/26/27/28/29* as promising candidate genes for further exploring how tobacco responds to *R. solanacearum* infection.

## Discussion

In this study, 37 *NtSTP* genes were identified (**Figure 1**). The larger number of STP paralogs in tobacco relative to diploid *A. thaliana* and *O. sativa* is consistent with gene family expansion triggered by allopolyploidization (Zan et al., 2025). Phylogenetic analysis classified these proteins into six Groups (I-VI), revealing a close evolutionary relationship between tobacco and *A. thaliana*, particularly within Group VI, which contained the highest number of orthologs (**Figure 3**). Notably, Subgroup V comprised exclusively NtSTP and AtSTP members, suggesting species-specific functional divergence in this clade. It is noteworthy that Group V within the NtSTP phylogeny exhibited low bootstrap support. This phenomenon is frequently encountered in large gene families subjected to frequent tandem duplications, such as the STP family. The high degree of sequence conservation across the transmembrane domains, coupled with relatively recent duplication events, results in extremely short internal branches. This “compression” of evolutionary time limits the number of parsimony-informative sites, leading to phylogenetic uncertainty regarding the exact sister-group relationships. Therefore, while the assignment of these genes to their respective subfamilies is statistically sound, the specific branching order within these tight clusters should be interpreted with caution.

All 37 NtSTP proteins contained the core MFS_STP domain, confirming their identity as sugar transporters. However, the exon-intron architecture varied significantly across subgroups. For instance, members of Group V exhibited a simple intronless structure, whereas those in Groups I, III, and IV displayed more complex gene structures with 3–5 exons. This structural diversity likely contributes to functional specialization, as seen in other plant species (Liu et al., 2022; Huang et al., 2025). The conserved motif and gene structure analyses further corroborate the reliability of the phylogenetic classification. Members within the same clade shared highly similar motif compositions, and the presence of core motifs across most NtSTP proteins suggests that these elements underpin the fundamental transport function of the family (**Figure 4**). Conversely, the absence of specific motifs in certain members indicates functional divergence, a pattern consistent with the evolutionary dynamics observed in other plant species (Liu et al., 2018; Qin et al., 2024). Notably, gene structure analysis revealed extreme simplification within Group V, where members predominantly consist of a single exon. This contrasts sharply with the more complex architecture (3–5 exons) observed in other subgroups. This is similar to the *STP* gene structure of *A. thaliana* (Büttner, 2010), *O. sativa* (Toyofuku et al., 2000) and *V. vinifera* (Afoufa-Bastien et al., 2010). This might be the result of variations such as the deletion or insertion of introns and exons in the *STP* gene structure during evolution, leading to the differentiation of the gene structure.

The gene duplication and Ka/Ks analysis indicated that tandem duplication was the core driving force for the expansion of the *NtSTP* family, and the Ka/Ks values of all duplicated gene pairs were less than 1 (**Figure 5**), indicating that the evolutionary process of this family was mainly dominated by purifying selection, effectively eliminating harmful mutations and maintaining the stability of gene functions. This is highly similar to the evolutionary selection pattern of the STP family in *M. esculenta*, *B. oleracea* and *O. sativa* (Toyofuku et al., 2000; Liu et al., 2018; Zhang et al., 2019). The interspecific collinearity analysis showed that the STP family of *N. tabacum* was more homologous to that of the same family of dicotyledons (*S. lycopersicum*) than to that of monocotyledons (*O. sativa*), further confirming that the evolution of the *STP* family has obvious species phylogenetic conservatism (Toyofuku et al., 2000; Liu et al., 2018; Zhang et al., 2019; Qin et al., 2024).

The analysis of the cis-acting elements of the promoter revealed that the *NtSTP* family promoters are enriched with a large number of hormone response, biological and non-biological stress, light signal, and growth and development regulatory elements. They also contain abundant binding sites for transcription factors such as MYB, MYC, and W-box, confirming that the *NtSTP* genes are subject to the coordinated regulation by endogenous hormones and external environmental signals, possessing the molecular basis for participating in multiple stress responses. This is consistent with the regulatory characteristics of the *STP* family gene in *T. aestivum* (Liu et al., 2021a) and *B. oleracea* (Zhang et al., 2019). Combined with the RT-qPCR verification results of the stress caused by *R. solanacearum* infection, *NtSTP1*, *NtSTP5*, *NtSTP7*, *NtSTP21*, *NtSTP22*, *NtSTP24*, *NtSTP26*, *NtSTP28*, and *NtSTP29* genes can significantly respond to *R. solanacearum* infection (**Figure 7**). Some members do not show significant changes in expression. This indicates that the *NtSTP* family has significant functional differentiation in *N. tabacum R. solanacearum* defense, which is consistent with the functional characteristics of *B. oleracea* (Zhang et al., 2019) and *C. Calla* (Huang et al., 2025) *STP* genes involved in bacterial disease defense. This provides key candidate genes for deciphering the molecular mechanism of *N. tabacum* resistance to *R. solanacearum* infection.

The induction of these *NtSTP* genes during *R. solanacearum* infection implies that they might participate in reshaping sugar distribution and plant immune responses. *R. solanacearum* is a vascular pathogen that depends on apoplastic sugars to support its proliferation. On this basis, we put forward a tentative hypothesis aligned with the nutritional immunity framework: upregulated *NtSTPs* could enable the host to reduce sugar supply in the apoplast and thereby restrict bacterial multiplication (Ke et al., 2023). Importantly, this mechanistic scenario remains speculative in the absence of direct quantification of apoplastic sugar levels and heterologous functional characterization of these sugar transporters. This is supported by studies in wheat, where *TaSTP3* and *TaSTP13* are associated with susceptibility to rust fungi by regulating sugar supply to the pathogen (Huai et al., 2020, 2022). Similarly, in *A. thaliana*, *AtSTP1* and *AtSTP4* contribute to stomatal movement by transporting glucose into guard cells, influencing pathogen entry (Truernit et al., 1996; Sherson et al., 2000; Otori et al., 2019). Although direct evidence for sugar transport inhibition by *NtSTPs* in *N. tabacum* is lacking, the coordinated upregulation of multiple *NtSTP* genes under infection strongly implies a role in rerouting carbon flux toward defense pathways. Furthermore, the enrichment of hormone-responsive elements in *NtSTP* promoters suggests that their expression is modulated by defense-related hormones. For example, the induction of *NtSTP21* and *NtSTP29* may be linked to jasmonic acid (JA) and salicylic acid (SA) signaling, which are central to plant immunity against vascular pathogens (Chen et al., 2024b; Liu et al., 2025b). The presence of TCA-element and CGTCA-motifs also hints at cross-talk between abiotic stress responses and biotic defense (**Figure 7**), as observed in *T. aestivum* (Liu et al., 2021b). And NtSTP21 and AtSTP3 were clustered in the same subgroup, which further confirmed this speculation (**Figure 3**).

This study is the first to conduct a comprehensive genome-wide systematic analysis of the *N. tabacum STP* family, clarifying its evolutionary characteristics, structural diversity, transcriptional regulatory patterns, and the response to *R. solanacearum*. This study offers preliminary theoretical support for subsequent molecular breeding applications and in-depth functional mechanism exploration of *N. tabacum STP* genes. However, this study still has certain limitations: only through bioinformatics prediction and transcriptional expression analysis was the disease resistance potential of the *NtSTP* family preliminarily clarified. No functional verification experiments such as gene overexpression and gene editing were carried out, and the specific molecular mechanism of key genes regulating bacterial wilt resistance could not be elucidated. In addition, the upstream transcription factors regulating the *NtSTP* genes were not screened, and the core regulatory pathway could not be clarified. Future research should prioritize the functional characterization of key disease-resistance candidate genes to unravel the molecular mechanisms and signaling networks underlying tobacco resistance to bacterial wilt. In this study, genome-wide identification and structural annotation of *NtSTP* genes were carried out using the published NtaSR1 v1.0 reference genome (cv. Petite Havana SR1), whereas all RT-qPCR expression experiments were performed on the widely cultivated tobacco cultivar K326. Genetic variations such as single nucleotide polymorphisms (SNPs) and insertions/deletions (InDels) exist between different tobacco cultivars, which could lead to minor inconsistencies in gene sequences or gene identifiers between the SR1 reference and the K326 genetic background. Although SR1 and K326 are both *Nicotiana tabacum* and harbor highly conserved STP coding sequences and functional domains, this cultivar mismatch may result in subtle divergence in gene sequences and transcriptional regulation. Nevertheless, the expression profiles generated from K326 remain biologically credible for evaluating stress responses of *STP* family genes. Further research utilizing a K326 self-matched reference genome will facilitate more precise gene annotation and mechanistic analysis.

## Conclusions

This study presents the first comprehensive genome-wide analysis of the *NtSTP* gene family in *N. tabacum*, identifying 37 members that were classified into six Groups. Our analyses revealed that tandem duplication (TD) played a dominant role in gene family expansion under strong purifying selection, whereas structural divergence, exemplified by the intron-less architecture of Group V genes, may have contributed to their functional diversification during evolution. Promoter analysis revealed a complex regulatory network involving hormone signaling (ABA, JA, SA) and stress responses. Notably, *NtSTP1*/*5*/*7*/*21*/*22*/*24*/*26*/*27*/*28*/*29* were significantly induced by *R. solanacearum* infection, exhibiting distinct temporal dynamics. These expression signatures suggest that particular *NtSTP* paralogs undergo refined transcriptional reprogramming and may modulate sugar distribution during tobacco responses to bacterial wilt. This study generates valuable genetic resources for subsequent functional research and offers a basis to further investigate *STP* activity in plant stress responses.

## Data Availability

The original contributions presented in the study are included in the article/**Supplementary Material**. Further inquiries can be directed to the corresponding author.
