# Supplementary material for: Genome-wide characterization of the sugar transporter protein family identifies candidate genes for bacterial wilt resistance breeding in tobacco
Source: Front Plant Sci. 2026 Aug 31;17:1937657. doi: 10.3389/fpls.2026.1937657 (PMC13573574; doi:10.3389/fpls.2026.1937657)
Supplement: Supplementary file 1 [file Table2.doc]

Figure S1. The phylogenetic trees were generated of 37 NtSTP, 28 OsSTP, 21 ZmSTP and 14 AtSTP proteins by MEGA 12.1 (method: Maximum Likelihood (ML: bootstrap values of 1000 replicates). Magenta, purple, orange, red, green and blue represents Group I, Group II, Group III, Group IV, Group V and Group VI, respectively.
